# In vivo PET imaging of neuroinflammation in familial frontotemporal dementia

**DOI:** 10.1136/jnnp-2020-323698

**Published:** 2020-10-29

**Authors:** Maura Malpetti, Timothy Rittman, Peter Simon Jones, Thomas Edmund Cope, Luca Passamonti, William Richard Bevan-Jones, Karalyn Patterson, Tim D Fryer, Young T Hong, Franklin I Aigbirhio, John Tiernan O'Brien, James Benedict Rowe

**Affiliations:** 1 Department of Clinical Neurosciences, and Cambridge University Hospitals NHS Trust, University of Cambridge, Cambridge, Cambridgeshire, UK; 2 Istituto di Bioimmagini e Fisiologia Molecolare Consiglio Nazionale delle Ricerche, Milan, Italy; 3 Department of Psychiatry, University of Cambridge, Cambridge, Cambridgeshire, UK; 4 Wolfson Brain Imaging Centre, University of Cambridge, Cambridge, Cambridgeshire, UK; 5 Medical Research Council Cognition and Brain Sciences Unit, Cambridge, Cambridgeshire, UK

**Keywords:** frontotemporal dementia, PET, genetics

## Abstract

**Introduction:**

We report *in vivo* patterns of neuroinflammation and abnormal protein aggregation in seven cases of familial frontotemporal dementia (FTD) with mutations in MAPT, GRN and C9orf72 genes.

**Methods:**

Using positron emission tomography (PET), we explored the association of the distribution of activated microglia, as measured by the radioligand [^11^C]PK11195, and the regional distribution of tau or TDP-43 pathology, indexed using the radioligand [^18^F]AV-1451. The familial FTD PET data were compared with healthy controls.

**Results:**

Patients with familial FTD across all mutation groups showed increased [^11^C]PK11195 binding predominantly in frontotemporal regions, with additional regions showing abnormalities in individuals. Patients with MAPT mutations had a consistent distribution of [^18^F]AV-1451 binding across the brain, with heterogeneous distributions among carriers of GRN and C9orf72 mutations.

**Discussion:**

This case series suggests that neuroinflammation is part of the pathophysiology of familial FTD, warranting further consideration of immunomodulatory therapies for disease modification and prevention.

## Introduction

A fifth of frontotemporal dementia (FTD) cases are autosomal dominant,^1^ most commonly arising from mutations in MAPT, GRN or C9orf72 genes.^2^ The pathological and clinical features of familial FTD closely resemble sporadic cases, but mutation carriers allow inference of the underlying pathology with molecular specificity. Although misfolding and aggregation of tau or TDP-43 (43 kDa transactive response DNA binding protein) protein leads to characteristic FTD neuropathology, neuroinflammation may be an early aetiopathogenic process, rather than a consequence of neurodegeneration. This hypothesis is supported by genome-wide association studies that implicate immunological pathways in FTD,^3^ and animals studies that identified inflammatory changes preceding tau accumulation.^4^ In addition, positron emission tomography (PET) with the radioligand [^11^C]PK11195 reveals in vivo neuroinflammation in frontotemporal regions in FTD,^5 6^ even before the onset of symptoms in genetic cases.^7^ In presymptomatic FTD associated with a MAPT mutation, [^11^C]PK11195 PET revealed increased levels of microglial activation, despite the lack of significant atrophy or binding of [^18^F]AV-1451,^7^ a radioligand sensitive to the presence of tau and TDP-43 pathology. However, the presence and pattern of inflammation by genotype, and its relationship to phenotype and to tau/TDP-43 burden, remain underinvestigated.

We used [^11^C]PK11195 to quantify *in vivo* neuroinflammation in patients with symptomatic familial FTD from MAPT, GRN or C9orf72 mutations. We then compared the distribution of inflammation to the distribution of [^18^F]AV-1451 binding.

## Methods

Seven patients with familial FTD were recruited from the Cambridge University Centre for Frontotemporal Dementia: two with MAPT 10+16 gene mutation, two with GRN C388_391delCAGTp.(Gln130fs) and three with C9orf72 expansions. Six patients met diagnostic criteria for behavioural variant FTD (bvFTD)^8^ and one for non-fluent variant primary progressive aphasia (nfvPPA).^9^ The demographics of the cohort are given in online supplemental table 1 and the clinical features of each patient are described in detail in the Results section alongside the imaging findings. Six of the seven patients with familial FTD were included alongside 25 sporadic FTD cases in a previous publication using different analyses,^6^ but the following analysis differs in several key respects including (i) separate comparison of each genetic case versus controls, without principal component analysis of the group data; (ii) voxel-wise analysis rather than only regional analysis. They underwent a structured clinical and neuropsychological assessment, together with brain imaging with 3T MRI, [^11^C]PK11195 PET (also called (*R*)-[^11^C]PK11195, from the ^11^C-labelled R-enantiomer of PK11195) and [^18^F]AV-1451 PET.^10^

10.1136/jnnp-2020-323698.supp1 Supplementary data

Structural MRI, [^11^C]PK11195 PET and [^18^F]AV-1451 PET data were acquired and processed as previously described.^6 10^ To quantify the density of radioligand binding, non-displaceable binding potential (BP_ND_) was calculated for both [^11^C]PK11195 and [^18^F]AV-1451 at the voxel level and also in 83 regions of interest (ROIs) based on a modified version of the n30r87 Hammersmith atlas (http://www.brain-development.org), with ROI data corrected for cerebrospinal fluid (CSF) partial volume prior to kinetic modelling.

To examine the [^11^C]PK11195 and [^18^F]AV-1451 binding distributions at the single subject level, we employed three approaches. First, we visually evaluated the BP_ND_ maps for each patient, in relation to their symptoms, as commonly applied for qualitative assessment in clinical practice. Second, for each radioligand we calculated voxel-wise Z-score maps by comparing BP_ND_ for each patient versus a group of healthy controls to identify voxels with significantly increased BP_ND_. Prior to determination of Z-scores, each BP_ND_ map was first warped to ICBM 152 2009a space using parameters from the spatial normalisation of the coregistered T1 MR image of each subject, then masked to reduce the impact of extracerebral signal and smoothed (isotropic 6 mm full width at half maximum Gaussian). In view of substantial off-target subcortical [^18^F]AV-1451 binding, we confine our visual inspection of the [^18^F]AV-1451 BP_ND_ and Z-score maps to cortical regions. Finally, as a secondary quantitative analysis, one-tailed Crawford tests for single-case analysis^11^ were performed on CSF-corrected regional BP_ND_ values for each radioligand to test for significant differences between each case and controls. For the voxel-wise and regional comparisons between patients and controls, we used two age-matched and sex-matched groups of healthy elderly adults (online supplemental table 2), who underwent either [^11^C]PK11195 PET (N=15) or [^18^F]AV-1451 PET (N=15).

## Results

**Figure 1:**
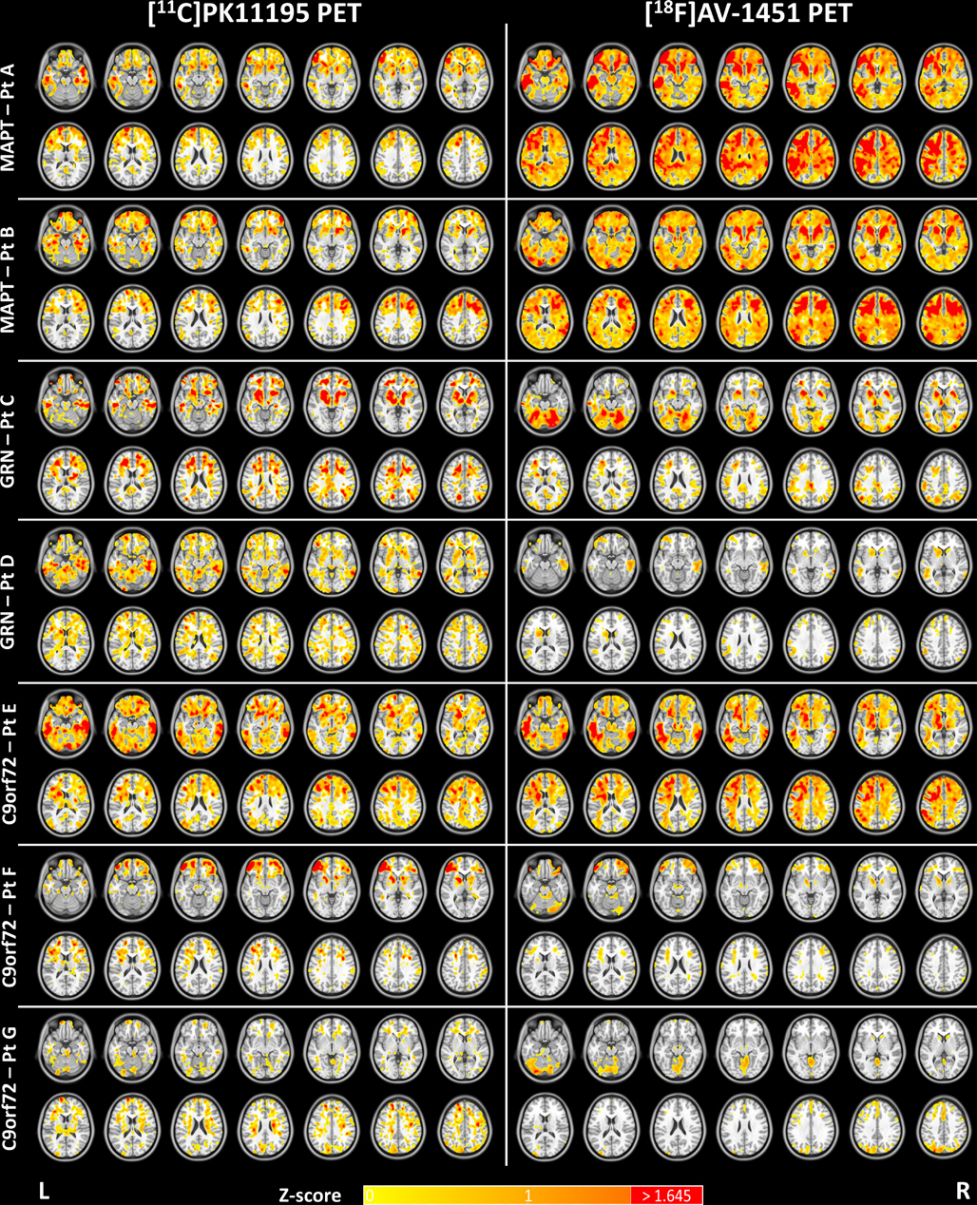
Z-score maps for [^11^C]PK11195 (left panel) and [^18^F]AV-1451 (right panel) binding potential for each patient (A–G) as compared with 15 controls. Patients A and B are MAPT mutation carriers; cases C and D are patients with GRN mutations; and patients E, F and G are C9orf72 mutation carriers. The Z-score scale applies to both PET tracers (Z-scores>1.645 which corresponds to p<0.05 are reported in red). The slices are reported in the neurological display convention (left on the left) and the Z-score maps are overlaid on the ICBM 152 2009a T1 MR template. Pt, patient; PET, positron emission tomography.

For each familial FTD patient BP_ND_ maps for [^11^C]PK11195 and [^18^F]AV-1451 are presented in online supplemental figure 1, alongside the corresponding mean BP_ND_ map for controls. Z-score maps for each patient and radioligand are shown in figure 1, while regional comparisons for [^11^C]PK11195 and [^18^F]AV-1451 are reported in online supplemental table 3 and online supplemental table 4, respectively.

### MAPT mutations

*Patient A* was diagnosed with bvFTD aged 51, with progressive lack of insight and language, impairment of memory and judgement, loss of functional skills, apathy and loss of semantic knowledge. She scored 43/100 on the Addenbrooke’s Cognitive Examination Revised (ACE-R), scoring 0/14 at the fluency test, 9/18 on the Frontal Assessment Battery (FAB), 2/30 on the Frontotemporal Dementia Rating Scale (FRS) and 118/180 on the Cambridge Behavioural Inventory Revised (CBI-R). She had a family history of FTD, with her father carrying a symptomatic MAPT 10+16 mutation. [^11^C]PK11195 binding was elevated in the medial and superior temporal lobes and temporal poles, the medial and posterior orbitofrontal cortex, the medial and dorsolateral prefrontal cortex, the cingulate gyrus, subcortical structures (ie, globus pallidus, putamen and nucleus accumbens) and mildly in parietal regions. Elevated [^18^F]AV-1451 binding overlapped with [^11^C]PK11195 and it was spatially more extensive, involving extensively the frontal and temporal lobes, especially the anterior and medial temporal regions, the orbitofrontal cortex and the dorsolateral prefrontal cortex. The cingulate cortex and the inferior parietal lobe also showed increased [^18^F]AV-1451 binding. For some frontal regions and temporal regions, both [^11^C]PK11195 and [^18^F]AV-1451 binding were more elevated in the left hemisphere.

*Patient B* presented at 60 years of age with bvFTD, characterised by obsessional and compulsive behaviours, comprehension impairments, anomia and a positive family history of dementia consistent with FTD. She scored 44/100 on ACE-R, 7/18 on FAB, 1/30 on FRS and 99/180 on CBI-R. PET scans showed increased [^11^C]PK11195 binding mainly in the anterior and medial temporal regions, the fusiform gyri and the middle-inferior frontal cortex. Secondarily, the dorsolateral and medial prefrontal cortex, the right parietal lobe and subcortical structures (ie, putamen and nucleus accumbens) were also involved in the elevated [^11^C]PK11195 binding distribution. Similar to Patient A, the pattern of increased [^18^F]AV-1451 binding overlapped with that of [^11^C]PK11195, but was more extensive than the latter. Increased [^18^F]AV-1451 binding was evident across all frontal lobes, mainly in dorsolateral prefrontal cortex, middle frontal gyrus, anterior cingulate gyrus and orbitofrontal regions. Milder increased [^18^F]AV-1451 binding was also seen in medial temporal and parietal regions.

### GRN mutations

*Patient C* presented bvFTD at age 70 with apathy, emotional incontinence and disinhibition, but no motor signs. She scored 33/100 on ACE-R, 3/30 on FRS, and 88/180 on CBI-R. [^11^C]PK11195 binding was increased in the inferior temporal regions, orbitofrontal cortex and subcortical structures (ie, putamen, globus pallidum and substantia nigra). Increased [^11^C]PK11195 binding was also patchily seen in the medial prefrontal cortex and parietal lobes. [^18^F]AV-1451 PET showed mildly increased binding in the left medial and inferior temporal regions. The Z-score map showed increased [^18^F]AV-1451 levels in cerebellum, however this was not highlighted by the regional comparisons with controls.

*Patient D* presented progressive non-fluent aphasia at age 65 with additional mild behavioural changes including jocularity and hoarding of sweets. He was diagnosed with nfvPPA 18 months after his symptom onset. By the time of the PET scans, he had declined significantly with minimal speech, yes/no confusion and significant behavioural problems without motor involvement. He scored 76/100 on ACE-R, 9/18 on FAB, 12/30 on FRS and 62/180 on CBI-R. There was no significant increased binding of [^18^F]AV-1451 at the regional level as compared with controls, with low intensity clusters seen on the Z-score map in the left inferior temporal lobe and the cerebellum. In contrast, the [^11^C]PK11195 Z-score map showed more extensive increased binding in the temporal lobes (anterior, medial, inferior and fusiform regions), and in the prefrontal cortex, especially in the left inferior frontal gyrus.

### C9orf72 mutations

*Patient E* presented bvFTD aged 56 years, with irritable behaviour, over-valued ideas and obsessions, a sweet tooth, semantic impairment, but no signs of motor neuron disease. He scored 53/100 on ACE-R, 6/18 on FAB, 9/30 on FRS and 76/180 on CBI-R. On PET imaging there was increased [^11^C]PK11195 binding widely in the temporal and frontal lobes, especially involving the anterior medial and lateral temporal regions and the orbitofrontal cortex. Scattered foci of increased [^11^C]PK11195 binding were seen in parietal lobes and cerebellum, however this was not significant at the regional level. There was increased [^18^F]AV-1451 binding in the anterior lateral and medial temporal regions most notably in the left hemisphere, and in the superior and middle frontal cortex. Milder uptake was seen also in left parietal cortex.

*Patient F* presented bvFTD at age 51 with behavioural changes, abnormal beliefs, stereotypical and repetitive behaviours, but no features of motor neuron disease. She developed paranoid delusions, apathy, a sweet tooth with significant weight gain, and a moderate aphasia with non-fluent speech and anomia. She scored 41/100 on ACE-R, 7/18 on FAB, 3/30 on FRS and 60/180 on CBI-R. Her pattern of [^18^F]AV-1451 binding was low across the whole-brain, and only very mild elevated binding in the lateral orbitofrontal regions. [^11^C]PK11195 PET showed increased binding in frontotemporal and parietal regions, with peaks in the orbitofrontal cortex, the anterior temporal lobes, the middle frontal regions and the left inferior frontal gyrus.

*Patient G* presented bvFTD at age 58 with a long history of psychiatric symptoms and an episode of psychotic depression at age 44 following bereavements. At age 56, he had a relapsing episode of depression with psychotic features and he was consequently treated with risperidone after which he developed a parkinsonian syndrome. His psychosis progressed and he developed disinhibited social behaviours and memory problems. There was a positive family history for motor neuron disease, although he had no signs or symptoms of motor neuron diseases. He scored 46/100 on ACE-R, 5/18 on FAB, 2/30 on FRS and next of kin endorsed 140/180 items on CBI-R. [^11^C]PK11195 PET showed scattered increases in binding in superior frontal and parietal regions, inferior temporal regions and cerebellum. There was no significant increased binding of [^18^F]AV-1451 at the regional level, while at the voxel level mildly increased [^18^F]AV-1451 binding was seen in parietal–occipital regions, cingulate cortex and cerebellum.

## Discussion

The study demonstrates *in vivo* neuroinflammation with the three most common monogenic forms of FTD. Familial FTD was associated with neuroinflammation in frontotemporal regions, across all genes.

The distribution of neuroinflammation might reflect clinical heterogeneity. For example, in both MAPT patients, [^11^C]PK11195 binding was elevated in fronto-striatal regions in association with behavioural variant FTD,^12^ but Patient A’s language impairments accompany increased binding in temporal regions. The GRN cases had different phenotypes for which the bvFTD in Patient C accompanied prominent orbitofrontal inflammation, but focal left inferior frontal inflammation in Patient D was associated with non-fluent aphasia. While inflammation in orbitofrontal regions may be associated with the neuropsychiatric symptomatology reported in the three C9orf72 cases, Patients E and F also presented language impairments, and increased inflammation in left inferior frontal gyrus and temporal regions. In these latter patients, who are expected to have TDP-43 pathology but not significant tauopathy, [^18^F]AV-1451 binding was not intense or extensive compared with the distribution of neuroinflammation ([^18^F]AV-1451 binding in the C9orf72 carriers was less marked than in semantic dementia, which typically has another form of TPD-43 pathology^13^).

Our findings align with previous evidence that support the importance of neuroinflammation in FTD.^14^ We suggest that activated microglia play an important role in defining clinical syndromes associated with genetic FTD-related mutations. Previous postmortem evidence in FTD reported upregulated microglial activation in both tau and TDP-related pathologies.^15^ In patients with MAPT mutations, neuroinflammation may be triggered by the misfolding of mutant tau and contribute to its hyperphosphorylation and aggregation,^4 14 16^ while in C9orf72 and GRN cases, neuroinflammation could be triggered by dipeptide repeats or progranulin haploinsufficiency in advance of the large TDP-43 aggregates.^14 17^ However, further studies with presymptomatic cases at risk of familial FTD will be needed to reveal the temporal association between microglial activation and specific patterns of protein aggregation. Such studies will also be needed to confirm gene-specific inflammation patterns and the consistency of their association with specific clinical syndromes.

We present both voxel-wise and regional analyses. Voxel-wise maps at single subject level may reveal local abnormalities, while regional comparisons focus on consistent and diffuse increases in radioligand binding. The ROI-based approach is more common in PET imaging, especially for group studies, given that it is more statistically robust. However, averaging the tracer uptake over a region can mask the focal binding intensities within the region. Thus, the methods are complementary, and the Z-score maps and ROI data are available on request for further comparison.

The case report approach is limited by its small size, which does not permit correlational analyses between PET imaging and clinical severity by genetic subgroups. However, small sample sizes can be informative about typicality of a feature in a population, when features are identified in the majority of cases sampled.^18^ To empower the ROI-based comparisons between each patient and controls, we included separate groups of healthy adults who underwent either [^11^C]PK11195 PET or [^18^F]AV-1451 PET but not both. However, the control groups were older than patients on average, and the increased binding levels in symptomatic cases may therefore be underestimated. In addition, [^11^C]PK11195 enables the visualisation of increased Translocator Protein (TSPO) expression, which is a marker for microglial activation. However, neuroinflammatory cascade in neurodegenerative diseases is a complex process not confined to activated microglia.^14 19^ Further studies will be needed to clarify other inflammatory processes associated with genetic FTD.

In conclusion, we suggest that regional inflammation is a typical feature of genetic FTD and future disease-modifying treatment strategies may be enhanced by immunomodulation. [^11^C]PK11195 PET studies in presymptomatic carriers could help develop early, targeted, interventions.
