# Supplementary material for: In vivo PET imaging of neuroinflammation in familial frontotemporal dementia
Source: J Neurol Neurosurg Psychiatry. 2020 Oct 29;92(3):319–22. doi: 10.1136/jnnp-2020-323698 (PMC7892378; doi:10.1136/jnnp-2020-323698)

Supplementary Material

Supplementary Table 1. Demographics, genetic and clinical features for each patient.

Pt	Gene	Diagnosis	Sex	Age at PET	Age at Diagnosis	Age at Onset	Disease duration at PET	Years of Education	ACE-R /100
**A**	MAPT	bvFTD	F	51	51	46	5.5	16	43
**B**	MAPT	bvFTD	F	61	60	52	9	16	44
**C**	GRN	bvFTD	F	71	70	66	4.8	10	33
**D**	GRN	nfPPA	M	66	65	63	2.4	10	76
**E**	C9orf72	bvFTD	M	56	56	54	2.8	10	53
**F**	C9orf72	bvFTD	F	51	51	47	4.5	10	41
**G**	C9orf72	bvFTD	M	59	58	56	3	9	46

Abbreviations: Pt=patient; bvFTD=behavioural variant frontotemporal dementia; nfPPA=non-fluent primary progressive aphasia; F=Female; M=Male; ACE-R= Addenbrooke's Cognitive Examination Revised

Supplementary Table 2. Demographics of the two age- and sex-matched control groups compared to each patient in radioligand-specific tests. Age, years of education, and ACE-R scores were compared between the two groups of controls with independent-samples t-tests, while sex was compared with the Chi-square test.

Group control	N	Sex (F/M)	Age (mean ± SD)	Education (mean ± SD)	ACE-R (mean ± SD)
**[^{11}C]PK11195**	15	8/7	68.8±5.5	14.4±2.8	93.3±4.4
**[^{18}F]AV-1451**	15	7/8	67.3±7.6	15.5±2.3	95.7±3.2
**Difference controls (p-value)**	**-**	0.72	0.56	0.26	0.11

Abbreviations: F/M= female/male; SD=standard deviation; ACE-R= Addenbrooke's Cognitive Examination Revised

Malpetti M, *et al. J Neurol Neurosurg Psychiatry* 2021; 92:319–322. doi: 10.1136/jnnp-2020-323698

Supplementary Table 3. [^{11}C]PK11195 binding potential (BP_{ND}) values and Z-scores (Z) for brain regions with statistically significant increased BP_{ND} in each patient (Pt) compared to controls at $p < 0.05$ uncorrected (tests surviving FDR correction are in bold). Mean and standard deviation (SD) BP_{ND} values for controls are also reported.

Region		Controls BP_{ND}		Pt A		Pt B		Pt C		Pt D		Pt E		Pt F		Pt G	
Name	#	Mean	SD	BP_{ND}	Z	BP_{ND}	Z	BP_{ND}	Z	BP_{ND}	Z	BP_{ND}	Z	BP_{ND}	Z	BP_{ND}	Z
Hippocampus R	1	0.01	0.07	0.28	**3.81**	-	-	-	-	-	-	-	-	-	-	-	-
Hippocampus L	2	0.04	0.07	-	-	0.29	**3.75**	-	-	-	-	-	-	-	-	-	-
Amygdala R	3	0.01	0.07	0.37	**4.95**	0.21	2.72	-	-	-	-	0.22	2.87	-	-	-	-
Amygdala L	4	0.06	0.04	0.34	**6.35**	0.34	**6.40**	-	-	-	-	-	-	-	-	-	-
Anterior temporal lobe medial part R	5	0.04	0.06	0.23	**3.11**	-	-	-	-	-	-	0.35	**5.10**	0.19	2.50	-	-
Anterior temporal lobe medial part L	6	0.04	0.06	0.25	**3.14**	0.22	2.68	-	-	-	-	-	-	-	-	-	-
Anterior temporal lobe lateral part R	7	0.10	0.07	0.26	2.42	0.33	**3.46**	-	-	-	-	-	-	-	-	-	-
Anterior temporal lobe lateral part L	8	0.03	0.05	0.27	**4.70**	0.26	**4.38**	-	-	-	-	0.19	3.11	0.27	**4.70**	-	-
Parahippocampal and ambient gyri R	9	0.05	0.05	0.27	**4.08**	0.19	2.56	-	-	-	-	0.22	3.05	-	-	-	-
Parahippocampal and ambient gyri L	10	0.07	0.05	0.28	**3.79**	0.41	**6.23**	-	-	-	-	0.22	2.73	-	-	-	-
Middle and inferior temporal gyrus R	13	0.01	0.04	-	-	0.14	**3.32**	-	-	0.11	2.56	-	-	-	-	-	-
Middle and inferior temporal gyrus L	14	-0.02	0.03	0.14	**6.02**	0.08	**3.77**	0.08	**3.70**	-	-	0.08	**3.95**	0.10	**4.50**	-	-
Fusiform gyrus R	15	-0.01	0.02	0.11	**5.07**	0.20	**9.27**	0.05	2.41	0.19	**8.68**	0.16	**7.42**	0.09	**4.14**	-	-
Fusiform gyrus L	16	-0.01	0.05	0.16	**3.36**	0.24	**4.84**	-	-	0.10	2.21	0.37	**7.33**	-	-	0.11	2.37
Insula L	20	0.02	0.05	-	-	-	-	-	-	-	-	0.16	2.92	-	-	-	-
Middle frontal gyrus L	28	-0.05	0.06	-	-	-	-	-	-	-	-	-	-	0.09	2.28	-	-
Middle frontal gyrus R	29	-0.01	0.05	-	-	0.08	1.84	-	-	-	-	-	-	0.11	2.63	-	-
Nucleus accumbens L	36	0.11	0.07	-	-	0.33	**3.06**	-	-	-	-	-	-	-	-	-	-
Nucleus accumbens R	37	0.08	0.08	0.45	**4.84**	-	-	-	-	-	-	-	-	-	-	-	-
Putamen L	38	0.06	0.05	-	-	-	-	0.29	**4.85**	0.16	1.98	-	-	-	-	-	-
Putamen R	39	0.08	0.04	0.15	1.83	0.18	2.59	0.19	3.07	0.15	1.98	-	-	-	-	-	-
Pallidum L	42	0.03	0.10	0.27	2.40	-	-	0.25	2.13	-	-	-	-	0.27	2.40	-	-
Straight gyrus L	52	0.18	0.07	0.43	**3.55**	-	-	-	-	-	-	-	-	-	-	-	-
Straight gyrus R	53	0.16	0.09	0.44	**3.20**	-	-	-	-	-	-	-	-	-	-	-	-
Anterior orbital gyrus L	54	0.04	0.06	-	-	-	-	-	-	-	-	-	-	0.37	**5.27**	-	-
Anterior orbital gyrus R	55	0.07	0.06	-	-	-	-	-	-	-	-	-	-	0.27	**3.15**	0.23	2.56
Inferior frontal gyrus L	56	0.09	0.05	-	-	-	-	-	-	-	-	-	-	0.23	2.83	-	-
Inferior frontal gyrus R	57	0.12	0.06	-	-	0.25	2.31	-	-	-	-	-	-	0.23	2.03	-	-
Superior frontal gyrus L	58	0.07	0.05	-	-	-	-	-	-	-	-	-	-	-	-	0.18	2.48
Superior frontal gyrus R	59	0.04	0.06	-	-	-	-	-	-	-	-	-	-	-	-	0.17	2.11

Lingual gyrus L	64	0.15	0.07	-	-	-	-	-	-	-	-	-	-	-	-	0.29	2.10
Lingual gyrus R	65	0.11	0.06	-	-	-	-	-	-	-	-	-	-	-	-	0.23	1.96
Cuneus L	66	0.17	0.05	-	-	-	-	-	-	-	-	-	-	0.35	**3.74**	0.27	2.12
Cuneus R	67	0.12	0.05	-	-	-	-	-	-	-	-	-	-	-	-	0.23	1.88
Medial orbital gyrus L	68	0.12	0.06	0.25	1.97	-	-	-	-	-	-	-	-	0.36	**3.82**	-	-
Lateral orbital gyrus L	70	0.13	0.06	-	-	-	-	0.28	2.42	-	-	-	-	0.52	**6.22**	-	-
Lateral orbital gyrus R	71	0.13	0.06	-	-	-	-	-	-	-	-	-	-	0.52	**6.27**	-	-
Posterior orbital gyrus L	72	0.10	0.07	0.29	2.55	-	-	-	-	-	-	-	-	-	-	-	-
Posterior orbital gyrus R	73	0.14	0.06	-	-	-	-	-	-	-	-	-	-	0.27	2.32	-	-
Substantia nigra L	74	0.22	0.07	-	-	-	-	-	-	-	-	-	-	0.44	2.91	-	-
Substantia nigra R	75	0.15	0.07	-	-	-	-	0.60	**6.47**	-	-	-	-	-	-	-	-
Subgenual frontal cortex L	76	0.01	0.07	0.30	**4.13**	-	-	-	-	-	-	-	-	-	-	-	-
Subcallosal area L	78	0.17	0.12	-	-	-	-	-	-	-	-	0.43	2.10	-	-	-	-
Subcallosal area R	79	0.23	0.16	-	-	-	-	0.68	2.91	-	-	-	-	-	-	-	-
Presubgenual frontal cortex L	80	0.15	0.10	0.35	1.87	-	-	-	-	-	-	-	-	-	-	-	-
Presubgenual frontal cortex R	81	0.10	0.08	0.25	1.83	-	-	-	-	-	-	-	-	-	-	-	-
Superior temporal gyrus anterior part L	82	0.09	0.09	0.32	2.53	-	-	-	-	-	-	-	-	-	-	-	-
Superior temporal gyrus anterior part R	83	0.11	0.10	0.29	1.93	0.30	1.99	-	-	-	-	0.35	2.48	-	-	-	-

Abbreviations: L=Left; R=Right

Supplementary Table 4. [^{18}F]AV1451 binding potential (BP_{ND}) values and Z-scores (Z) for brain regions with statistically significant increased BP_{ND} in each patient (Pt) compared to controls at $p < 0.05$ uncorrected (tests surviving FDR correction are in bold). Mean and standard deviation (SD) BP_{ND} values for controls are also reported.

Region		Controls BP_{ND}		Pt A		Pt B		Pt C		Pt D		Pt E		Pt F		Pt G	
Name	#	Mean	SD	BP_{ND}	Z	BP_{ND}	Z	BP_{ND}	Z	BP_{ND}	Z	BP_{ND}	Z	BP_{ND}	Z	BP_{ND}	Z
Hippocampus L	2	0.07	0.08	-	-	-	-	0.34	3.12	-	-	-	-	-	-	-	-
Amygdala R	3	0.06	0.07	0.35	**4.11**	-	-	-	-	-	-	-	-	-	-	-	-
Amygdala L	4	0.05	0.08	0.24	**2.51**	-	-	-	-	-	-	-	-	-	-	-	-
Anterior temporal lobe medial part R	5	0.00	0.06	0.25	**4.39**	0.14	2.42	-	-	-	-	0.15	2.59	-	-	-	-
Anterior temporal lobe medial part L	6	0.01	0.06	0.37	**6.62**	-	-	0.11	1.86	-	-	0.27	**4.80**	-	-	-	-
Anterior temporal lobe lateral part R	7	0.05	0.08	0.35	**3.67**	0.24	2.27	-	-	-	-	0.22	2.03	-	-	-	-
Anterior temporal lobe lateral part L	8	0.04	0.08	0.42	**4.85**	0.21	2.20	-	-	-	-	0.32	**3.55**	-	-	-	-
Parahippocampal and ambient gyri R	9	0.03	0.06	0.29	**4.11**	0.16	2.11	-	-	-	-	-	-	-	-	-	-
Parahippocampal and ambient gyri L	10	0.04	0.06	0.27	**3.66**	0.19	2.37	0.17	2.17	-	-	-	-	-	-	-	-
Middle and inferior temporal gyrus R	13	0.01	0.06	0.16	**2.56**	0.17	2.68	-	-	-	-	0.12	1.82	-	-	-	-
Middle and inferior temporal gyrus L	14	0.01	0.05	0.29	**6.01**	0.13	2.67	0.15	3.04	-	-	0.28	**5.83**	-	-	-	-
Fusiform gyrus R	15	0.02	0.08	0.30	**3.63**	0.21	2.43	-	-	-	-	-	-	-	-	-	-
Fusiform gyrus L	16	0.03	0.06	0.34	**5.60**	-	-	-	-	-	-	0.27	**4.36**	-	-	-	-
Insula L	20	0.02	0.05	0.15	2.41	-	-	-	-	-	-	-	-	-	-	-	-
Lateral remainder of occipital lobe L	22	-0.04	0.06	-	-	0.09	2.04	-	-	-	-	-	-	-	-	-	-
Cingulate gyrus anterior part L	24	0.13	0.06	0.27	2.32	-	-	-	-	-	-	-	-	-	-	-	-
Cingulate gyrus anterior part R	25	0.09	0.05	-	-	0.19	1.84	-	-	-	-	-	-	-	-	-	-
Cingulate gyrus posterior part L	26	0.07	0.05	0.16	1.84	0.18	2.17	-	-	-	-	-	-	-	-	-	-
Cingulate gyrus posterior part R	27	0.05	0.05	0.15	1.91	-	-	-	-	-	-	-	-	-	-	-	-
Middle frontal gyrus L	28	-0.06	0.05	0.06	2.29	0.16	**4.22**	-	-	-	-	0.07	2.58	-	-	-	-
Middle frontal gyrus R	29	-0.02	0.06	-	-	0.20	3.57	-	-	-	-	-	-	-	-	-	-
Posterior temporal lobe L	30	-0.01	0.06	0.13	2.33	0.13	2.32	-	-	-	-	-	-	-	-	-	-
Posterior temporal lobe R	31	-0.01	0.05	0.09	1.95	0.11	2.48	-	-	-	-	-	-	-	-	-	-
Inferiolateral remainder of parietal lobe L	32	-0.01	0.07	0.13	2.16	0.16	2.69	-	-	-	-	-	-	-	-	-	-
Inferiolateral remainder of parietal lobe R	33	0.01	0.05	-	-	0.16	3.10	-	-	-	-	-	-	-	-	-	-
Caudate nucleus L	34	0.21	0.10	0.40	1.85	-	-	-	-	-	-	-	-	-	-	-	-
Caudate nucleus R	35	0.18	0.11	-	-	0.43	2.34	-	-	-	-	-	-	-	-	-	-
Nucleus accumbens L	36	0.16	0.07	0.62	**6.35**	0.30	1.99	0.43	3.78	-	-	-	-	-	-	-	-

Malpetti M, *et al. J Neurol Neurosurg Psychiatry* 2021; 92:319–322. doi: 10.1136/jnnp-2020-323698

Nucleus accumbens R	37	0.19	0.12	0.42	1.92	0.42	1.86	-	-	-	-	-	-	-	-	-	-
Putamen L	38	0.26	0.08	-	-	0.40	1.85	-	-	-	-	-	-	-	-	-	-
Thalamus L	40	0.23	0.06	0.34	1.84	-	-	0.39	2.59	-	-	-	-	-	-	-	-
Pallidum L	42	0.18	0.09	0.42	**2.64**	0.39	2.34	-	-	-	-	0.45	2.97	-	-	-	-
Pallidum R	43	0.21	0.13	-	-	0.47	2.03	-	-	-	-	-	-	-	-	-	-
Straight gyrus L	52	0.15	0.07	0.50	**4.72**	-	-	-	-	-	-	-	-	-	-	-	-
Straight gyrus R	53	0.13	0.07	0.38	**3.68**	-	-	-	-	-	-	-	-	-	-	-	-
Anterior orbital gyrus L	54	0.01	0.07	0.15	1.92	-	-	-	-	-	-	-	-	-	-	-	-
Anterior orbital gyrus R	55	0.01	0.06	0.15	2.18	-	-	-	-	-	-	-	-	0.22	3.34	-	-
Inferior frontal gyrus L	56	0.06	0.06	0.22	**2.87**	0.23	3.03	-	-	-	-	-	-	-	-	-	-
Inferior frontal gyrus R	57	0.09	0.07	-	-	0.27	2.70	-	-	-	-	-	-	-	-	-	-
Superior frontal gyrus L	58	0.04	0.05	0.20	**2.94**	0.15	2.11	-	-	-	-	0.17	2.46	-	-	-	-
Superior frontal gyrus R	59	0.04	0.07	-	-	0.23	2.95	-	-	-	-	-	-	-	-	-	-
Superior parietal gyrus L	62	0.01	0.06	-	-	0.15	2.48	-	-	-	-	-	-	-	-	-	-
Medial orbital gyrus L	68	0.08	0.07	0.30	**3.35**	-	-	-	-	-	-	-	-	-	-	-	-
Medial orbital gyrus R	69	0.08	0.08	0.24	2.11	0.26	2.25	-	-	-	-	-	-	-	-	-	-
Lateral orbital gyrus L	70	0.05	0.07	0.36	**4.50**	0.18	1.87	-	-	-	-	-	-	0.29	3.43	-	-
Lateral orbital gyrus R	71	0.04	0.07	0.19	1.99	-	-	-	-	-	-	-	-	0.34	3.97	-	-
Posterior orbital gyrus L	72	0.04	0.05	0.33	**5.26**	-	-	-	-	-	-	-	-	-	-	-	-
Posterior orbital gyrus R	73	0.07	0.08	0.30	**2.79**	-	-	-	-	-	-	-	-	-	-	-	-
Substantia nigra L	74	0.26	0.11	-	-	-	-	-	-	-	-	0.54	2.56	-	-	-	-
Substantia nigra R	75	0.22	0.13	0.49	2.13	-	-	-	-	-	-	-	-	-	-	-	-
Subgenual frontal cortex L	76	-0.01	0.08	0.24	**3.09**	-	-	-	-	-	-	-	-	-	-	-	-
Subgenual frontal cortex R	77	-0.05	0.05	0.12	**3.49**	0.06	2.30	-	-	-	-	-	-	-	-	-	-
Subcallosal area L	78	0.14	0.12	0.39	2.05	-	-	0.47	2.63	-	-	-	-	-	-	-	-
Subcallosal area R	79	0.11	0.08	0.31	2.35	0.34	2.79	-	-	-	-	-	-	-	-	-	-
Presubgenual frontal cortex L	80	0.11	0.09	0.32	2.29	-	-	-	-	-	-	-	-	-	-	-	-
Presubgenual frontal cortex R	81	0.02	0.07	0.19	2.24	0.25	3.10	-	-	-	-	-	-	-	-	-	-
Superior temporal gyrus anterior part L	82	0.08	0.08	0.38	**3.72**	-	-	-	-	-	-	0.39	**3.87**	-	-	-	-

Abbreviations: L=Left; R=Right

Supplementary Figure 1. Axial slices of the [^{11}C]PK11195 (left) and [^{18}F]AV-1451 (right) binding potential (BP_{ND}) maps for each patient (A-G). For comparison, the last row shows the corresponding average BP_{ND} maps across 15 controls. Patients A and B are MAPT mutation carriers; cases C and D are patients with GRN mutations; and patients E, F and G are C9orf72 mutation carriers. The BP_{ND} maps were spatially normalised to ICBM 152 2009a space, masked and smoothed (isotropic 6mm full width at half maximum Gaussian). The BP_{ND} maps are overlaid on the ICBM 152 2009a T1 MR template and the slices are reported in the neurological display convention (left on the left). The BP_{ND} scale applies to both radioligands.

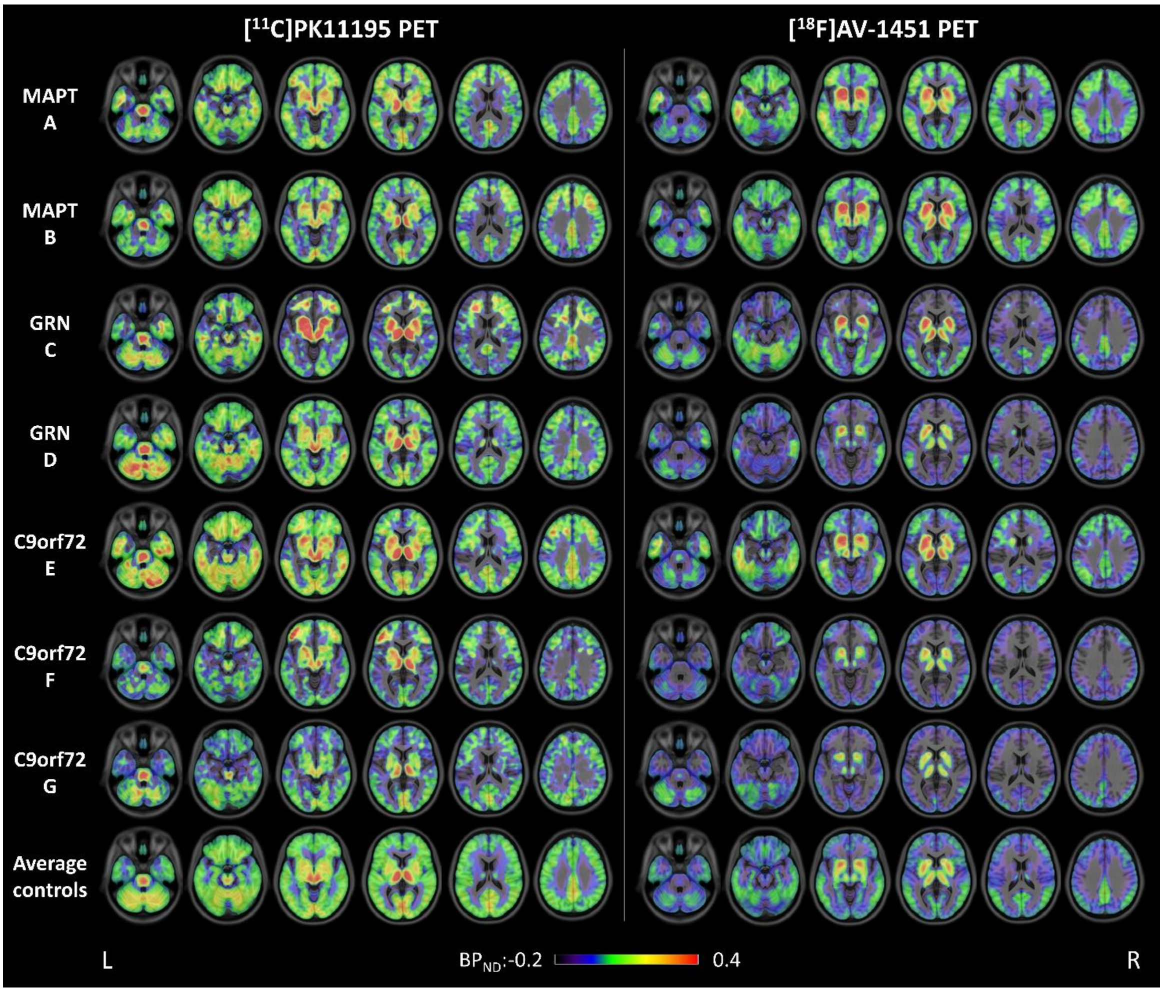

Supplement: Supplementary data [file jnnp-2020-323698supp001.pdf]
